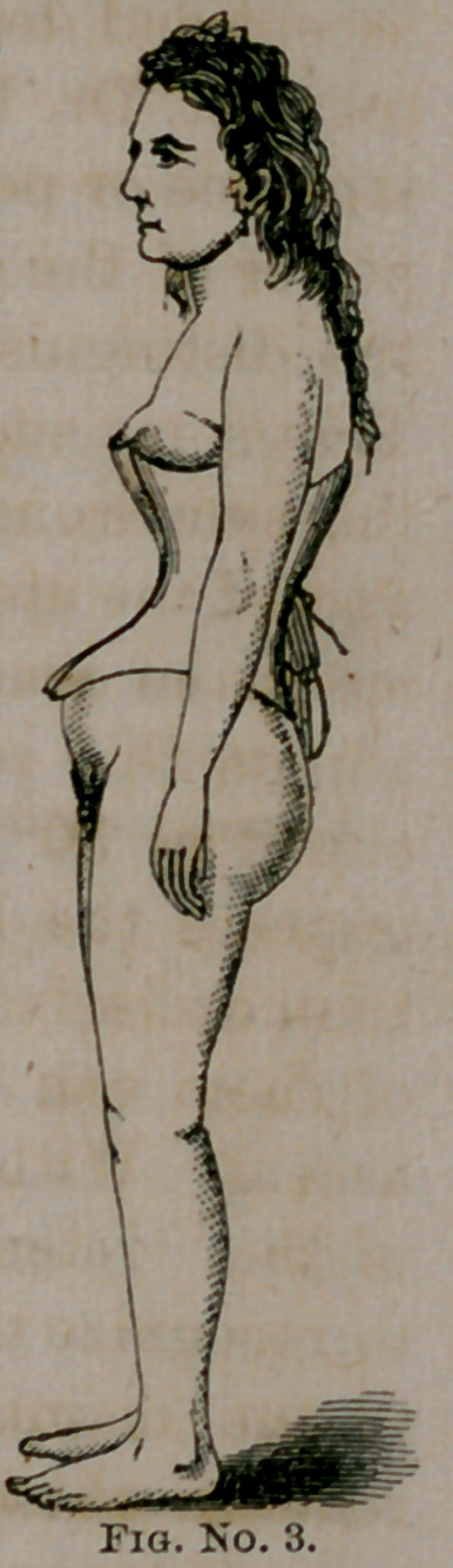# The Corset in Its Relations to Uterine Diseases

**Published:** 1873-03

**Authors:** V. H. Taliaferro

**Affiliations:** Professor of Diseases of Women in the Atlanta Medical College, Atlanta, Georgia


ATLANTA

### MEDICAL AND SURGICAL JOURNAL.

VOL. X.—MARCH, 1873.—NO. 12.

---

### ORIGINAL COMMUNICATIONS.

---

### THE CORSET IN ITS RELATIONS TO UTERINE DISEASES.

BY V. H. TALIAFERRO, M.D.,

*Professor of Diseases of Women in the Atlanta Medical College, Atlanta, Georgia.*

The improved corset, very aptly termed *glove-fitting*, is made of stout, heavy cloth, firmly starched, ribbed with steel and whalebone, and fitted (as if moulded) to the lower two-thirds of the thorax and the upper two-thirds of the abdomen—thus constituting a firm, close and perfectly adapted splint to the muscles it embraces. No fact in surgery is better established than that muscles long subjected to the action of splints, though *loosely applied*, become atrophied, and lose correspondingly in tone and strength. This is fully illustrated by the complaints of weakness, etc., in the disabled muscles which have been acted upon, in the case of any habitual corset wearer, who may be induced to attend to her accustomed duties or pleasures, for a day or two, without her corset.

Upon the vigor and tone of the *thoracic muscles* depend in a large degree the tone and vigor of the *thoracic viscera.* These muscles contribute largely to the promotion of general muscular strength and vigor, and, in conjunction with the diaphragm, *immediately to lung power*, and hence to the heart's action and to the functions of digestion and assimilation. It is in the lower part of the thorax that its great expansibility resides. The utmost freedom of this expansibility is absolutely essential to the healthful and uninterrupted action of the lungs. The direct effect of the corset upon this part of the thorax is to weaken the muscles compressed, and to close in the *false ribs* upon the lungs, the diaphragm, the stomach and the liver—suppressing the respiratory movements of the ribs and thoracic muscles, and hence necessitating the forces of the respiratory act downward upon the pelvic organs through the contiguous abdominal viscera.

In his general description of the thorax, Cruvelhier makes use of the following language: "The effects of strong and permanent constriction are also manifest in a very evident manner in the alteration of the form of the thorax consequent upon the use of stays. This species of constriction effects principally the lower part of the chest; so that the fifth, sixth, seventh, eighth, ninth and tenth ribs are pressed forward and inward, because the length and flexibility of their cartilages allow them to yield readily; and all the viscera which correspond to this species of girdle, undergo a very marked alteration in their direction, and even in their figure and position." "In an old female, whose thorax was so contracted below as to present the appearance of a barrel, and bore witness to the use of a very tight corset, the cartilage of the seventh rib, on the right side, was in contact with that of the opposite rib; the ziphoid appendix was strongly depressed and pushed behind the cartilages of the seventh and eighth ribs, which touched each other."

In the normal condition of the thorax, the open space between the sternal cartilages of the tenth ribs will average in measurement at least eight inches; the same measurement for the eighth ribs will be six inches, while that just below the point of the ensiform cartilage will be three inches. In habitual corset wearers these measurements will be found to be reduced in proportion to the extent of pressure made by the lacing cords and the length of time they have been used, *from a partial to complete approximation* of the sternal cartilages of the false ribs.

It would be interesting to know the number of pounds pressure made by a corset applied *moderately tight*. (No lady ever admits to tight lacing.) I have no doubt that a calculation which would approximate the truth, would be astounding. It is not unusual for a young lady to call assistance to the strength of her own arms in lacing her corset. With the compound pulley power of the lacing cords, fifty or seventy pounds may be easily drawn. In many instances, I have no doubt, (of ball-room or party occasions,) a pressure is applied approximating in number of pounds the net weight of the body. It will readily be perceived that a large per cent. of this pressure from the corset is expended upon the pelvic organs, the sternal cartilages of the false ribs being approximate, or perhaps overlapped, pressing upward the diaphragm and lungs, and downward the stomach, liver and intestines, upon the lower abdominal walls and pelvic viscera. I know it is claimed by all ladies who wear corsets that they are worn "very loosely." If we will be permitted to insert our fingers *beneath* the corset, we will be astonished at their ideas of "very loosely." And, indeed, admitting that they are worn loosely, their evil effects nevertheless exist, though certainly in less degree. In the sitting posture, the one largely occupied by women, the pressure of the body forward upon the front stays and whalebones bend them in upon the stomach and intestines—forcing the viscera downward directly upon the fundus of the uterus, and thus pressing that organ downward through the vagina, or laying it across the pelvis in a flexed, retroverted or anteverted position. It will be perceived, from the anatomical relations of the viscera, that a *pressure* upon the stomach, directed down *upon the intestines*, can not be made without a corresponding *displacement of the womb*. The uterus, poised upon the upper extremity of the vagina, and imbedded in a cushion of elastic areola tissue, suffers no injury in itself or in its adjacent tissues from *temporary displacements;* but if, from whatsoever cause, it is *habitually and persistently* displaced, it must eventuate in congestions, effusions, hypertrophies, etc. That this persistent and habitual displacement does occur, as a rule, from the continued use of the corset, there is no question.

FIG. NO. 1.

Dr. Busey, of Washington, D. C., "On the Nature of Uterine Supports," in the *American Journal of Obstetrics*, for February, 1872, very truly states that "any permanent disturbance or destruction of the normal axial relations of the pelvis with the body, or with its viscera, or interruption of the natural correlation of the pelvic contents, become factors in the causation of uterine displacements." It will be seen by the diagrams that the normal axial relations of the pelvis with the abdominal viscera is interrupted by the corset, and from this alone, independent of the pressure just referred to, displacements of the uterus are induced.

In ıeference to the forces which govern the physiological ascent and descent of the uterus synchronous with the respiratory act, Dr. Busey says: "The important factor in the maintainance or perversion of these normal forces is the 'retentive power of the abdomen,' to which attention was first called by the distinguished Edinburgh obstetrician, J. Mathews Duncan. That some such power does exist seems obvious, or how explain the synchronous ascent and descent through its longitudinal axis of the uterus during respiration? The anatomical arrangement and construction of its ligaments and attachments, which admits this regular and constant movement along a plane inclined at 30° to the horizon—a movement which absolutely opposes the law of gravitation—precludes the supposition of their exclusive agency, inasmuch as the point of tension of no one of them can be reached either in this physiological descent or ascent. Without stopping to investigate the sources and nature of this 'retentive power,' it is sufficient for the present purpose to recognize the important part which the abdominal walls play in the maintainance of its integrity. But, apart from any relation which the relaxation of the abdominal walls—a necessary consequence of and increased by every recurring pregnancy—may bear to 'the retentive power of the abdomen,' the diminished action of the abdominal muscles, which is in proportion to the extent of distention and relaxation, favors the gravitation of the abdominal viscera, especially of the intestines. Hence, a new force may be brought into action, which, according to the direction of its action, may occasion, either alone or in synchronous coöperation with other new or perverted forces, uterine displacements. If the relaxed and protuberant walls permit descent of its visceral contents through the axis of the body, surely there must be augmented pressure upon the womb through its longitudinal axis."

It is clearly evident that whatever forces or influences acting upon the abdominal walls which weaken by distention and relaxation their "retentive power," greatly conduces to uterine displacements and diseases. We have seen that the immediate action of the corset upon the abdominal viscera is to force them downward upon the *abdominal walls* and pelvic contents, making tense and protuberant the lower abdomen, and, as we will presently see, increasing its measurement in circumference from two to four inches. Under such pressure and distention, the elastic muscles of the abdomen can not long retain their normal tone and resiliency, but will eventually yield to a permanent *flabby, weakened* and *pendulous* state — a condition, indeed, so characteristic of the old corset wearer, that it might well be designated as the *corset belly*. Very many young ladies of our highly civilized and fashionable age, of but sixteen or eighteen summers, have *protuberant, flabby* and *pendulant abdomens*, with which nature rarely encumbers and disfigures the healthy matron of a dozen successful pregnancies. And are we surprised, when *the child* of eight and ten must needs don *the corset*. Shame upon *the fashions* when they distort and disease the bodies which God has given us, created in His own image. Our wood-cut illustrations are faithful representations of the abdominal outlines *with* and *without* the corset. Nos. 2 and 3 are copies of photographs from nature. No. 1 is from a beautiful and elegantly executed diagram by my friend, Dr. Rauschenberg, of this city. These show us truthfully the inevitable changes in the abdominal outlines, and the corresponding interruption in the normal relations of the abdominal and pelvic viscera from the use of the corset. The young woman (the subject of our photographic illustrations) is twenty-eight years of age, and the mother of two children. She has never worn corsets, except upon special

Fig. No. 2.

Fig. No. 3.

occasions, and has not, therefore, the dilated and pendulous abdomen of the habitual corset wearer. Though she has borne two children and led a reckless life, she has firm, solid abdominal walls, with symmetrical outlines—the uterine organs normal in condition and perfectly maintained in *situ naturali.*

The following table, though not so large and complete as we would wish, will greatly aid to demonstrate the facts we wish to elucidate.

MRS. B.—AGE TWENTY-EIGHT; ONE CHILD; WEIGHT, ONE HUNDRED AND FIFTEEN LBS.

| | | |
|---|---|---|
| Measurement around the waist without the corset | 23 | inches. |
| " " " with corset | 17 | " |
| " " " reduced by corset | 6 | " |
| " " the lower abdomen without corset | 26 | " |
| " " " " with corset | 30½ | " |
| " " " " increased by corset | 4½ | " |
| " from ostium vaginæ to os tincæ, without corset | 4 | " |
| " " " " with corset | 2 | " |
| " " " " reduced by corset | 2 | " |

MRS. H. AGE TWENTY-FIVE; TWO CHILDREN; ONE MISCARRIAGE; WEIGHT, ONE HUNDRED AND THIRTY POUNDS.

| | | |
|---|---|---|
| Measurement around the waist without the corset | 26½ | inches. |
| " " " with corset | 21 | " |
| " " " reduced by corset | 5½ | " |
| " " the lower abdomen without corset | 32½ | " |
| " " " " with corset | 34 | " |
| " " " " increased by corset | 1½ | " |
| " from ostium vaginæ to os tincæ, without corset | 4 | " |
| " " " " with corset | 3¼ | " |
| " " " " reduced by corset | ¾ | " |

MISS ——. AGE SIXTEEN; NO CHILDREN; WEIGHT, ONE HUEDRED AND TWENTY-SEVEN.

| | | |
|---|---|---|
| Measurement around the waist without the corset | 26½ | inches. |
| " " " with corset | 22 | " |
| " " " reduced by corset | 4½ | " |
| " " the lower abdomen without corset | 31½ | " |
| " " " " with corset | 34¼ | " |
| " " " " increased by corset | 2¾ | " |

Uterine measurement not permitted.

MISS ——. AGE TWENTY-SIX; NO CHILDREN; WEIGHT, ONE HUNDRED AND TWENTY-FIVE.

| | | |
|---|---|---|
| Measurement around the waist without the corset | 26½ | inches. |
| " " " with corset | 21½ | " |
| " " " reduced by corset | 5 | " |
| " " the lower abdomen without corset | 34½ | " |
| " " " " with corset | 35½ | " |
| " " " " increased by corset | 1 | " |
| " from ostium vaginæ to os tincæ, without the corset | 3⅛ | " |
| " " " " with corset | 2⅛ | " |
| " " " " reduced by corset | 1 | " |

The first case, Mrs. B., had been an old corset wearer, though did not begin its use until after she was grown, and consequently did not, as yet, have any great permanent deformity of the thorax, though she had the genuine *corset belly.* She had been treated for endometritis with partial prolapsus, and relieved of all her disagreeable symptoms, except the leucorrhœa. This continued for months after the treatment was suspended, when she was induced to abandon her corset. Very soon thereafter her leucorrhœa ceased, and she has since had no return of it, some twelve months having elapsed. This result was obtained without further uterine treatment.

The second case, Mrs. H., had been under my treatment for some months previous to the measurements, and was still under treatment at that time for endometritis with some considerable parenchymatous congestion. I suspected her disease to be specific in its character. She had no displacement, and her disease yielded rapidly to treatment. She has never worn corsets except upon rare occasions, and but for a short while, as they were always disagreeable and painful to her. Although she had borne two children at full term, and had one miscarriage, she had firm and solid abdominal walls, without any undue bulging or protuberance.

The fourth case, Miss ——, had never borne children, and had never worn corsets but upon very few occasions. Her abdomen was perfect in form and symmetry. She had no uterine disease or displacement, and *never* had so far as she knew or suspected.

The difference in the measurements is accounted for by the difference in the conditicns of the abdominal walls and in the character of the resulting displacements. If there is simply a descent of the uterus, it will of course be greater than when the descent is attended with any degree of *flexion* or *version.*

The mobility of the uterus in all the cases examined was greatly impaired by applications of the corset, the organ being to a greater or less degree, for the time being, fixed in its abnormal position. Displacements of the uterus, when of short duration, are most likely attended with no evil or unpleasant results, but when repeated and continued from day to day, week to week, and from months to years, the injury will surely become profound and permanent.

"Displacements of the uterus at first results in passive congestion. This being kept up, hypergenesis of connective tissue takes place."—*Thomas.*

"It is (chronic engorgement) often developed after simple hyperæmia of the uterus, resulting from troubles of the circulation in the vessels of the pelvis."—*Scanzoni.*

"The causes of this diffuse growth of connective tissue must be sought for in habitual hyperæmia. It (diffuse growth of connective tissue) also occurs in many displacements of the uterus, especially those in which venous reflux is hindered." *Klob.*

It has been shown that the immediate result of the corset upon the uterus is *displacement.* We have seen, by the more recent and advanced uterine pathologists, that the circulatory disturbances consequent upon displacements result in a hyperæmia of the uterine vessels, and this in "diffuse growth of connective tissue"—proliferation—or hypertrophy of connective tissue, and consequent enlargement and induration of the uterus.

Displacements of the uterus occuring from the mechanical action of the corset, interferes directly, not alone with the circulation in the uterine plexus of veins, which imbed the walls of the uterus in a net-work of vessels, but the pressure from the crowding together of the pelvic viscera obstructs necessarily the return flow from the uterine tissues through the internal iliacs. The constriction around the waist and the consequent pressure upon the vena cava, contributes likewise to the coëxisting obstructed circulation in the uterine tissues. The vaginal, hemorrhoidal, ovarian and vesical veins are also implicated in greater or less degree in these circulatory disturbances; and hence we often have, complicating the uterine disease from this cause, ovarian, vesical, vaginal and rectal disorders. May we not also in this way explain some of the obscure hyperæsthesias of the female genito-urinary organs.

To sum up the result of our inquiries, we find that the habitual use of the corset contributes to the causation of uterine diseases by the following methods: First, interference by compression with the functions of digestion and respiration, and the consequent deterioration of the vital powers, thus predisposing to uterine diseases. Second, loss of "retentive power"

in the abdominal walls by distention and dilatation. Third, direct pressure upon and displacement of the uterus. Fourth, circulatory disturbances consequent upon the uterine displacement, and pressure upon the vena cava and the pelvic blood vessels.

I take pleasure in directing attention to a highly interesting communication from Dr. Frank Ramsey, of Tennessee, upon the importance of the *abdominal muscles* in uterine displacements. In this communication to the Gynæcological Society of Boston, he states:

"In this connection, I will urge upon the members of the Society the very great advantage there is possessed by all females who have been instructed to develop the abdominal muscles by use. In very many hundred females that have passed under my professional observation during the past thirty years, I have had no difficulty in destroying all inconveniences of ordinary displacements by the development of the muscles of the abdominal walls, and all muscles that could, by voluntary use, have any connection with the sexual organs."

While we would admit the temporary advantage, under some circumstances, of the *abdominal supporters* recommended by Dr. Thomas, in his admirable text book upon diseases of women, we must insist upon their deleterious effects when *habitually* worn—abdominal weakness and relaxation increasing necessarily under their constant use. Muscles assisted by supports, and hence obstructed in their action, must lose in tone and power. *Lifting, sweeping, making beds, etc.*, will give natural and permanent supports to the abdominal and pelvic viscera in *firm and strong abdominal muscles.* We would take occasion here to urge caution in exercise with the abdominal muscles and viscera impeded in their normal action by a corset. In the ordinary act of lifting, the abdominal muscles contract and force inward and upward the viscera—this being interrupted by the pressure of the corset from above, directs necessarily the force of the lift downward upon the pelvic viscera, and the already tense and distended lower abdomen. Hence, the disability with so many ladies to lift even small weights without inconvenience or injury to themselves; and hence also the frequent abortions, miscarriages, sudden displacements of the diseased or impregnated uterus, congestions, etc.

The following case may serve to illustrate the ultimate effects upon the uterine organs in a large number of cases in unmarried life:

Miss ——, some thirty years of age, came under my treatment pale and anæmic, and suffering with the long train of nervous sympathies incident to diseases of the uterus. She complained especially of defective menstruation, chronic dyesntery, leucorrhœa and headache. Her uterine troubles began to attract attention very soon after the establishment of the menstrual function. She knows of no existing cause or accident from which to attribute her troubles. They seemed to have increased perceptibly from year to year to the present time, when the general health has become greatly impaired. Physical examination reveals the hymen and vagina morbidly irritable and sensitive; the uterus high in the pelvis, with almost an entire absence of the vaginal cervix, from the stretching upward of the vaginal cul-de-sac. The fundus was found large, acutely anti-flexed, and easily felt above the pubis. The vagina was found red, congested, and highly sensitive. The sound gave three and three-eighth inches depth of uterine cavity, but little tenderness. At each menstrual period, the mammæ are painful and enlarged, and the bowel disturbance aggravated.

This lady has worn her corset from early girlhood. Her thorax is greatly deformed. The sternal cartilages of the false ribs are lapped over the ensiform cartilage, and are approximated in almost their entire extent. The *corset belly* is strikingly exemplified. She had also a peculiar *huskiness of voice* which may be frequently observed as a symptom attending uterine diseases, especially in the nullipara and in the virgin. We have in this case a *permanent and irremediable injury inflicted by the corset.* Her condition may be, and has already been, greatly improved by treatment, absence of the corset, loose clothes suspended from the shoulders, exercise, etc.

According to Graily Hewitt, the large plexuses of veins which issue from the sides of the uterus become "strangulated by flexions. A ligature is substantially applied to the uterus when it is flexed or bent upon itself." Such a condition can have no other result than congestion, hypertrophy, and induration. Such a condition we have in the case presented.

Acute congestions, or endometritis, and pelvic peritonitis, have often, I am sure, their cause in tightly laced corsets. As an example, we give the following case: In the fall of 1870, I was called to visit a young negro woman lately married, and supposed to be threatened with "miscarriage." I found a negro midwife in attendance upon the patient, stating that she had been with her during the night, constantly expecting that she would "get through." Upon inquiry as to the date of her pregnancy, etc., I found that she had not missed her regular menstrual periods, and that she was only thought to be in the "family way," because of the violent paroxysmal uterine pains. Upon a digital examination the uterus was found low in the pelvis, enlarged, almost immovable, and quite tender upon pressure, with considerable tenderness in the peri-uterine regions. Expressing to the patient and the midwife doubts as to pregnancy, I directed a dose of castor oil, to be followed in its action by opium and hot poultices over the hypogastrium, and instructed them to call on me again if she was not soon relieved. I should have stated that the woman's general health had previously been uninterrupted, and that I could elicit, upon inquiry, no cause to which to attribute an acute uterine attack. Upon my next visit, within some forty-eight hours, I found the patient much in the same condition, with perhaps an aggravation of all the symptoms. I now satisfied myself, by a thorough physical examination, that the case was one of acute congestion of the uterus, with acute perimetritis, or more properly, as Professor Thomas expresses it, *pelvic peritonitis.* Upon renewing my inquiries as to *the cause*, a friend and attendant of the sick woman, said to her, "Why don't you tell the Doctor what made you sick?—you know he ought to know it." I thus ascertained that she had dressed to go out Sunday, the day she was taken sick, and had worn a corset (to which she was unaccustomed) *laced very tightly*, and, after walking about a half mile, was seized with violent uterine pains and compelled to return home. It is unnecessary to give the details. She went through a painful and tedious acute uterine attack, from which she narrowly escaped with her life. *Cause and effect* are here as clearly evident as the history of any case can make them.

## Figures and Tables

**Fig. No. 1. f1:**
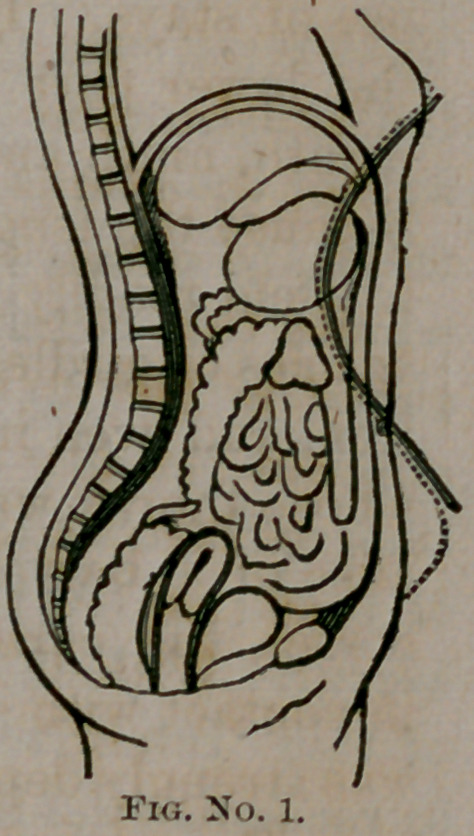


**Fig. No. 2. f2:**
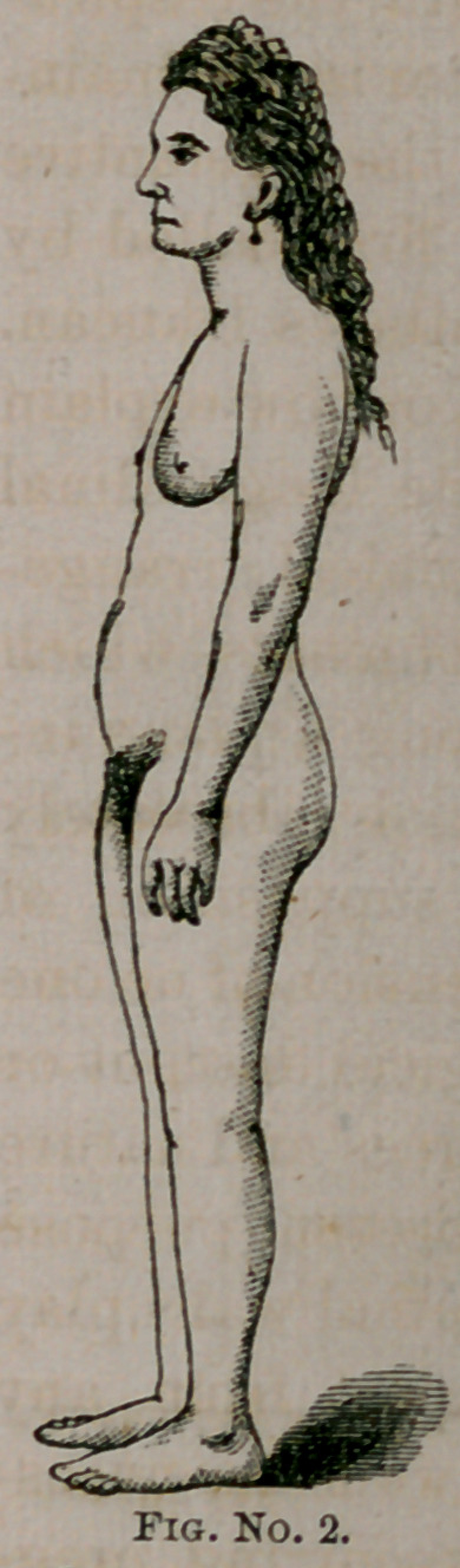


**Fig. No. 3. f3:**